# Substitute for Mercury in Syphilis

**Published:** 1853-03

**Authors:** 


*Substitute for Mercury in Syphilis.*—M. Robin lately brought before the Academy of Medicine of Paris, ten cases treated by M. Vicenti, which would prove the efficacy of bichrcmate of potash as an anti-syphilitic agent. Fom the facts, of which M. Robin gave a detailed account, he draws the following conclusions: 1. Bichromate of potash is now ascertained to be an anti-syphilitic agent. 2. The salt being very soluble, acts without loss in extremely small doses, the treatment being therefore shorter than when mercury is used. 3. Bichromate of potash does not in general produce salivation. 4. The only disadvantages hitherto noticed are nausea and vomiting when the salt is taken fasting; but these unpleasant effects do not take place when the medicine is administered a little time after the digestion of a meal, and especially when it is associated with opium. 5. It is of much use in neuralgia, and though it may produce asthenic effects, it is by no means deleterious. 6. Its exciting properties may render it nseful in indolent ulcers, in more or less strong solutions; as also in syphilitic sore throat, in the form of gargle. 7. As the ten patients who have taken the bichromate have not experienced the least unpleasant symptom, even by using very large doses for a protracted period, the new anti-syphilitic agent is now proved to be of greater value than the salts of mercury, which latter may become reduced in the economy, whilst the bichromate is irreducible under the same circumstances, and is so soluble as to be easily eliminated. Two of the above mentioned cases were treated in 1850 and 1851, and no kind of relapse has been noticed.—*Lancet.*

